# Neoantigen mRNA vaccines induce progenitor-exhausted T cells that support anti-PD-1 therapy in gastric cancer with peritoneal metastasis

**DOI:** 10.1007/s10120-025-01640-8

**Published:** 2025-07-31

**Authors:** Koji Nagaoka, Hideyuki Nakanishi, Hiroki Tanaka, Jessica Anindita, Takeshi Kawamura, Toshiya Tanaka, Takefumi Yamashita, Akihiro Kuroda, Sachiyo Nomura, Hidetaka Akita, Keiji Itaka, Tatsuhiko Kodama, Kazuhiro Kakimi

**Affiliations:** 1 Department of Immunology, Kindai University, Faculty of Medicine, 377-2 Ohnohigashi, Osakasayama, Osaka 589-0014 Japan; 2 Department of Biofunction Research, Laboratory for Biomaterials and Bioengineering, Institute of Integrated Research, Institute of Science Tokyo, 2-3-10 Kanda-Surugadai, Chiyoda-Ku, Tokyo, 101-0062 Japan; 3 Clinical Biotechnology Team, Center for Infectious Disease Education and Research (CiDER), The University of Osaka, 1-10 Yamadaoka, Suita, Osaka 565-0871 Japan; 4 Laboratory of DDS Design and Drug Disposition, Graduate School of Pharmaceutical Sciences, Tohoku University, 6-3 Aoba, Aramaki, Aoba-Ku, Sendai, 980-8578 Japan; 5 Isotope Science Center, The University of Tokyo, 2-11-16 Yayoi, Bunkyo-Ku, Tokyo 113-0032 Japan; 6 Laboratories for Systems Biology and Medicine, Research Center for Advanced Science and Technology, The University of Tokyo, 4-6-1 Komaba, Meguro-Ku, Tokyo, 153-8904 Japan; 7 Department of Physical Chemistry, School of Pharmacy and Pharmaceutical Sciences, Hoshi University, 2-4-41 Ebara, Shinagawa-Ku, Tokyo, 142-8501 Japan; 8 Department of Gastrointestinal Surgery, Graduate School of Medicine, The University of Tokyo, 7-3-1 Hongo, Bunkyo-Ku, Tokyo, 113-8655 Japan; 9 Department of Clinical Pharmaceutical Sciences, School of Pharmacy and Pharmaceutical Sciences, Hoshi University, 2-4-41 Ebara, Shinagawa-Ku, Tokyo, 142-8501 Japan

**Keywords:** Gastric cancer, Peritoneal metastasis, Neoantigen, mRNA vaccine, Immune checkpoint inhibitor

## Abstract

**Background:**

Gastric cancer with peritoneal metastasis is associated with a poor prognosis. Current treatments, including the first-line therapy of combination chemotherapy with nivolumab for advanced recurrent gastric cancer, have shown limited efficacy against peritoneal dissemination. In this study, we evaluated neoantigen (neoAg)-mRNA lipid nanoparticle (LNP) as a potential agent in combination with anti-PD-1 therapy, focusing on its effects on neoAg-specific CD8^+^ T cell responses and antitumor efficacy in a murine gastric cancer model.

**Methods:**

The mRNA, comprising a tandem minigene encoding three neoAgs identified from the murine gastric cancer YTN16 cell line, was synthesized by in vitro transcription and encapsulated within LNPs. NeoAg-specific CD8^+^ T cells in the spleens and tumors were assessed by flow cytometry. The antitumor efficacy of the neoAg-mRNA-LNP vaccine, alone or in combination with anti-PD-1 antibody, was evaluated in both subcutaneous and peritoneal metastasis models of YTN16.

**Results:**

The neoAg-mRNA-LNP vaccine induced significantly higher frequencies of neoAg-specific CD8^+^ T cells than the neoAg-dendritic cell vaccine, confirming its enhanced immunogenicity. NeoAg-mRNA-LNP vaccination led to robust tumor regression, achieving complete eradication in all treated mice, especially when combined with anti-PD-1 therapy. This effect was associated with an increase in neoAg-specific progenitor-exhausted and intermediate-exhausted CD8^+^ T cells. In a peritoneal metastasis model, neoAg-mRNA-LNP monotherapy prevented peritoneal dissemination when administered prophylactically, and combination therapy with anti-PD-1 effectively suppressed tumor growth in a therapeutic setting.

**Conclusions:**

NeoAg-mRNA-LNP vaccines elicit potent neoAg-specific CD8^+^ T cell responses and show enhanced antitumor efficacy with anti-PD-1 therapy in gastric cancer with peritoneal metastasis.

**Supplementary Information:**

The online version contains supplementary material available at 10.1007/s10120-025-01640-8.

## Introduction

In 2022, gastric cancer ranked fifth worldwide in both incidence and mortality, with approximately 970,000 new cases diagnosed and 660,000 deaths [1]. The peritoneal metastases are the most common form of recurrence after gastric cancer surgery [2], often leading to bowel obstruction and substantial ascites accumulation, and are associated with poor median survival, ranging from 4 to 6 months [3, 4].

For patients with gastric cancer accompanied by peritoneal metastases, hyperthermic intraperitoneal chemotherapy (HIPEC) combined with cytoreductive surgery (CRS) is considered a viable treatment option. Although several randomized phase III trials suggest that CRS + HIPEC extends overall survival (OS) [5, 6], a recent study has reported no significant OS benefit [7], underscoring ongoing debate regarding its efficacy.

Nivolumab combined with chemotherapy has been introduced as a first-line treatment option for advanced or metastatic gastric cancer based on the results of the global CheckMate 649 trial, which demonstrated significant improvements in both OS and progression-free survival (PFS), particularly among patients with high PD-L1 expression [8]. The ATTRACTION-4 trial demonstrated an extension in PFS with nivolumab plus chemotherapy; however, no significant efficacy was observed in gastric cancer patients with peritoneal metastases, as indicated by subgroup analysis [9]. Given the limited efficacy of nivolumab plus chemotherapy in gastric cancer patients with peritoneal metastasis, there is a critical need for combinatorial strategies that enhance the antitumor activity of anti-PD-1 therapies for this challenging condition.

Unlike tumor associated antigens such as the MAGE-A antigens, which are self-antigens subjected to central tolerance, neoantigens (neoAgs) are tumor-specific antigens arising from somatic mutations and are not expressed in normal tissues [10]. Because of their lack of central tolerance, neoAgs are recognized as truly non-self by the immune system and can elicit high-avidity CD8⁺ T cell responses. This makes neoAgs particularly attractive targets for cancer immunotherapy, as they are less likely to induce off-target immune responses and more likely to generate durable tumor-specific immunity.

We previously established the YTN16 cell line, a murine gastric cancer cell line capable of forming both subcutaneous and peritoneal dissemination models in immunocompetent C57BL/6 mice [11]. Additionally, we identified three neoAgs in YTN16 [12]. NeoAg peptide-pulsed dendritic cell (DC) vaccines induced a moderate level of neoAg-specific CD8^+^ T cells and demonstrated antitumor effects; however, these effects were not sufficient. Here, we investigated the efficacy of an mRNA vaccine targeting these neoAgs to evaluate its potential in combination with anti-PD-1 therapy to improve outcomes in gastric cancer with peritoneal metastases.

## Materials and methods

### Mice, cell lines and reagents

Six-week-old female C57BL/6N mice were purchased from Japan SLC (Shizuoka, Japan) and housed in a specific pathogen-free environment at the Center for Animal Experiment at Kindai University Faculty of Medicine. The animal use proposal and experimental protocol were reviewed and approved by the Kindai University Animal Committee (ID: KAME-2024–006). The YTN16 cell line was maintained in Dulbecco’s modified Eagle’s medium (DMEM, Nacalai Tesque, Kyoto, Japan) with 10% heat-inactivated fetal bovine serum (FBS, Sigma-Aldrich, St. Louis, MO, USA), 100 μg/mL streptomycin, 100 U/mL penicillin (Nacalai Tesque), and MITO + serum extender (Corning, Corning, NY, USA). The other reagents were listed in Supplementary Table 1.

### Preparation of neoAg-mRNA-lipid nanoparticle (LNP)

Preparation of mRNA-LNP was conducted based on a post-encapsulation method (manuscript under review). Details are described in Supplementary Materials and Methods.

### Vaccination and assessment of tumor growth

Naïve mice were vaccinated once or twice with either 1 μg or 5 μg of neoAg-mRNA-LNP or with 1 × 10^6^ neoAg-dendritic cells (DCs) via subcutaneous injection, with a two-week interval between doses. Two weeks after the last vaccination, splenocytes were analyzed using the MHC class I dimer assay or intracellular cytokine staining (ICS). To assess the antitumor activity of vaccine-induced T cells, mice were inoculated subcutaneously with 5 × 10^6^ YTN16 cells or intraperitoneally with 1 × 10^7^ YTN16-Luc cells. Subcutaneous tumor growth was monitored every 2–3 days using calipers and tumor volume was calculated using the formula π/6 × L1 × L2 × H, where L1 represents the long diameter, L2 the short diameter, and H the height of the tumor. Peritoneal dissemination of YTN16-Luc cells was monitored through bioluminescent imaging.

In the therapeutic experiment for subcutaneous tumors, mice were subcutaneously inoculated with 5 × 10^6^ YTN16 cells on day 0, followed by vaccination and/or injection of anti-PD-1 antibody on days 7 and 14, or on days 10 and 17. Tumor growth was monitored every 2–3 days. On day 21, the tumors were harvested and analyzed using the MHC class I dimer assay. In the therapeutic experiment for peritoneal metastases, mice were intraperitoneally injected with 1 × 10^7^ YTN16-Luc cells on day 0. The neoAg-mRNA-LNP vaccine and anti-PD-1 antibody were administered on days 7 and 14, and on days 7, 14, and 21, respectively. The anti-PD-1 antibody (200 μg) was administered via intraperitoneal injection.

### Statistical analysis

Statistical analyses were performed with Prism version 10.2.2. Comparisons were performed using Kruskal–Wallis test followed by Dunn’s multiple comparisons test or Mann–Whitney test. Data are presented as individual values and as the mean ± SD.

Additional methodological details are provided in the Supplementary Materials and Methods.

## Results

### neoAg-mRNA-LNP vaccines efficiently induce neoAg-specific CD8^+^ T cells compared with neoAg-DC vaccines.

**Fig. 1:**
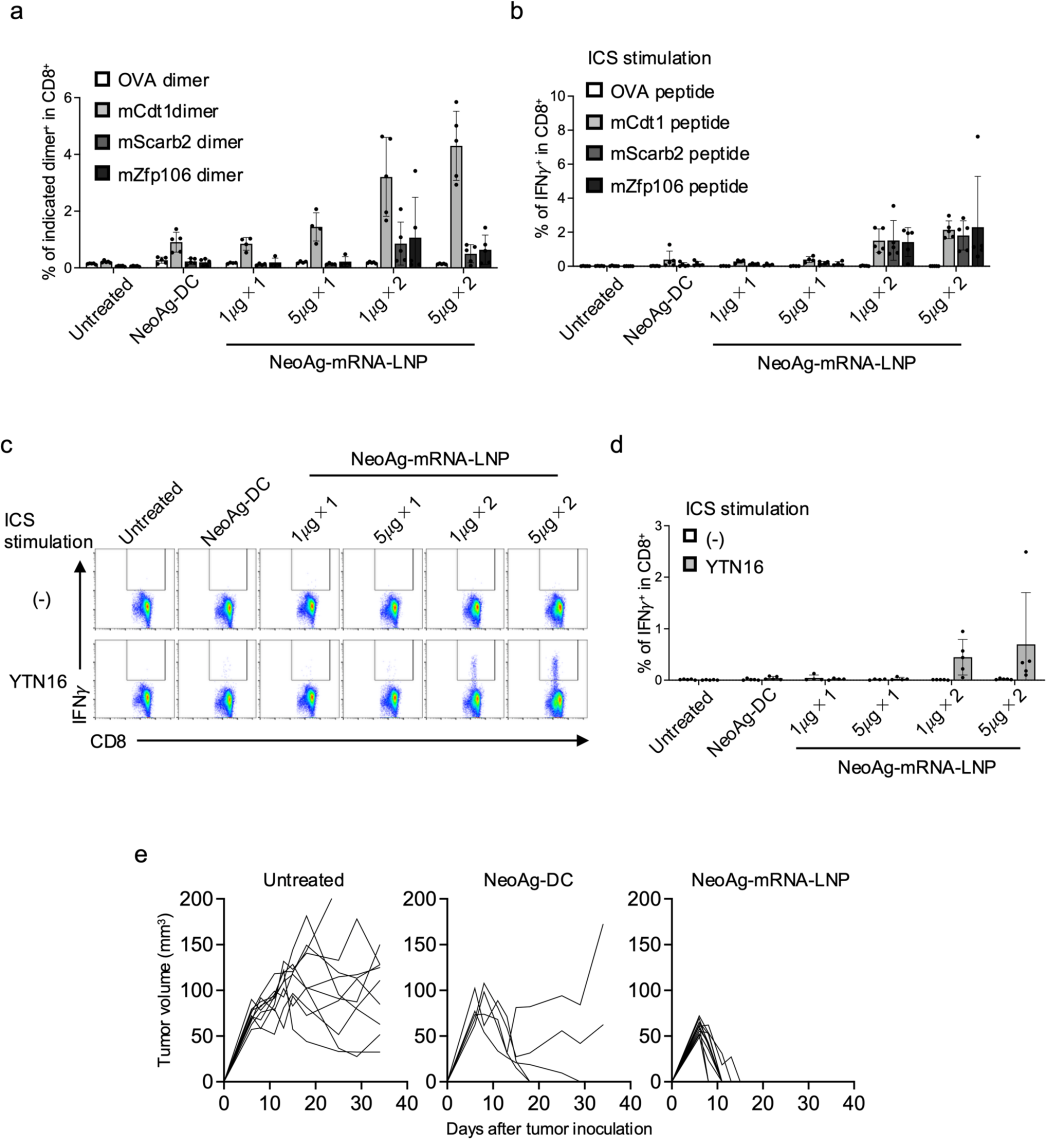
NeoAg-mRNA-LNP vaccines efficiently induce neoAg-specific CD8^+^ T cells capable of eradicating YTN16 tumor. **a**–**d** Naïve mice (*n* = 4 or 5 per group) were vaccinated with neoAg-DC-vaccines, 1 μg or 5 μg neoAg-mRNA-LNP vaccines once or twice at a 2-week-interval. Two weeks after the last vaccination, the splenocytes were subjected to MHC class I dimer assay (**a**) and intracellular cytokine staining (ICS) (**b**). **a** Bar graph shows the frequency of indicated dimer^+^ cells. Representative dot plots are shown in Supplementary Fig. 2a. **b** Bar graph shows the frequency of IFNγ–producing cells in CD8^+^ T cells in response to the indicated peptide. Representative dot plots are shown in Supplementary Fig. 2b. **c**, **d** The splenocytes were stimulated with YTN16 tumor cells for 4 h and stained with anti-CD8 and anti-IFNγ. **c** Dot plots show the expression of CD8 and IFNγ in CD8^+^ T cells. **d** Bar graph shows the frequency of IFNγ-producing cells in CD8^+^ T cells. **e** Naïve mice (*n* = 10, 5 and 12 for untreated, neoAg-DC and neoAg-mRNA-LNP groups, respectively) were vaccinated with neoAg-DC vaccines, or 5 μg neoAg-mRNA-LNP vaccines twice at a 2-week-interval. Two weeks after the second vaccination, 5 × 10^6^ YTN16 cells were inoculated subcutaneously. Tumor volumes were measured 2 or 3 times a week

We initially evaluated the induction of neoAg-specific CD8^+^ T cells by the neoAg-DC and neoAg-mRNA-LNP vaccines using three neoAgs that we had previously identified: mCdt1, mScarb2, and mZfp106 [12]. First, naïve mice were administered the DC vaccines twice, two weeks apart, and neoAg-specific CD8^+^ T cells in the spleen were analyzed two weeks after the second vaccination using an MHC class I dimer assay. The frequencies of mCdt1, mScarb2, and mZfp106-specific CD8^+^ T cells were 0.91 ± 0.35%, 0.23 ± 0.10%, and 0.20 ± 0.12%, respectively (Fig. 1a, Supplementary Fig. 2a), consistent with our previous findings [12]. Next, we examined the dose and administration frequency of the neoAg-mRNA-LNP vaccine. A single dose of 1 μg neoAg-mRNA-LNP vaccine induced a comparable level of neoAg-specific CD8^+^ T cells to the DC vaccine (Fig. 1a). The frequency of neoAg-specific CD8^+^ T cells increased with higher doses and multiple administrations. After two doses of the 5 μg neoAg-mRNA-LNP vaccine, neoAg-specific CD8^+^ T cells were induced at frequencies of 4.3 ± 1.2% for mCdt1, 0.50 ± 0.32% for mScarb2, and 0.64 ± 0.52% for mZfp106, demonstrating superior induction compared to the neoAg-DC vaccine. Similar results were observed in the ICS assay (Fig. 1b, Supplementary Fig. 2b). Notably, while the neoAg-DC vaccine induced only a minimal IFNγ response in CD8^+^ T cells upon stimulation with YTN16 cells, two doses of the mRNA-LNP vaccine, particularly at the 5 μg dose, led to robust IFNγ production by CD8^+^ T cells in response to YTN16 (Fig. 1c and d). These findings indicate that two doses of the neoAg-mRNA-LNP vaccine not only expanded T cells with neoAg-specific TCRs but also induced CD8^+^ T cells capable of recognizing MHC class I peptide complexes endogenously produced and presented by YTN16 cancer cells. Although the 5 μg dose appeared to induce slightly higher frequencies of neoAg-specific CD8⁺ T cells and greater IFNγ production than the 1 μg dose, the differences were relatively modest. However, both doses elicited substantially stronger responses than the DC vaccine. Given the absence of apparent adverse events at either dose and the consistently stronger immunogenicity observed with 5 μg, we proceeded to use this dose for subsequent experiments involving two administrations.

Next, we evaluated the antitumor effects of vaccine-induced neoAg-specific CD8^+^ T cells, including their role in tumor growth inhibition (Fig. 1e). Upon subcutaneous inoculation with YTN16 cells, all untreated mice developed tumors; however, 3 out of 5 mice receiving the neoAg-DC vaccine experienced tumor regression, while those vaccinated with the neoAg-mRNA-LNP achieved complete regression in 12 out of 12 mice. These findings demonstrated that the neoAg-mRNA-LNP vaccine induces a more robust population of tumor-reactive, neoAg-specific CD8^+^ T cells than the neoAg-DC vaccine, resulting in a stronger antitumor immune response.

### NeoAg-mRNA-LNP vaccine monotherapy and in combination with anti-PD-1 antibody exhibits strong antitumor effects in a therapeutic setting

**Fig. 2:**
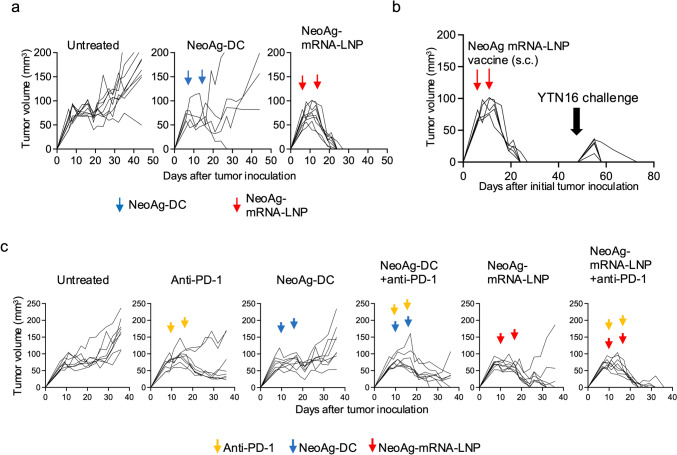
NeoAg-mRNA-LNP vaccines elicit robust antitumor responses in a therapeutic setting. **a** Mice (*n* = 9 for untreated and neoAg-mRNA-LNP groups and *n* = 5 for neoAg-DC group) were inoculated with 5 × 10^6^ YTN16 cells on day 0 and received two doses of vaccine on days 7 and 14. Tumor volumes were measured 2 or 3 times a week. Graphs show the tumor volumes of individual mice. **b** The mice that had rejected YTN16 by neoAg-mRNA-LNP vaccines (*n* = 5) were re-challenged with 5 × 10^6^ YTN16 cells on day 48. Graph shows the tumor growth of individual mice. **c** Mice (*n* = 8 per group) were inoculated with 5 × 10^6^ YTN16 cells on day 0 and received two doses of vaccine and/or anti-PD-1 mAb (200 μg) on days 10 and 17. Tumor volumes were measured 2 or 3 times a week. Graphs show the tumor growth of individual mice

Next, we conducted a therapeutic experiment in mice with pre-established subcutaneous YTN16 tumors, by administering vaccines on days 7 and 14 after tumor inoculation (Fig. 2a). While the neoAg-DC vaccine induced tumor regression in only 1 out of 5 mice, the neoAg-mRNA-LNP vaccine resulted in tumor regression in all 9 mice. Mice that had experienced tumor regression following neoAg-mRNA-LNP vaccination were re-challenged with YTN16 tumors approximately four weeks after the initial regression (Fig. 2b). Although tumors initially formed, they rapidly regressed in all 5 mice, indicating the formation of long-term memory in these tumor eradicated mice.

To establish a model in which the tumor is growing, and therapeutic effects are challenging to achieve, treatment was initiated 10 days after YTN16 tumor inoculation (Fig. 2c). While anti-PD-1 antibody monotherapy temporarily reduced tumor size, it did not lead to complete regression. The neoAg-DC vaccine monotherapy temporarily suppressed tumor growth; however, the tumors eventually resumed progressive growth. When neoAg-DC vaccine was combined with anti-PD-1, tumor regression was observed in 3 out of 8 mice. The neoAg-mRNA-LNP vaccine monotherapy led to tumor regression in 5 out of 8 mice, and when combined with anti-PD-1 antibody, tumor regression was observed in all 8 mice. These results indicate that while the neoAg-mRNA-LNP vaccine alone exhibits substantial antitumor activity, its combination with anti-PD-1 therapy further potentiates this effect, leading to complete tumor regression in all treated mice.

### Combination therapy of neoAg-mRNA-LNP vaccines and anti-PD-1 induced abundant neoAg-specific Tex^prog^ in the tumor

**Fig. 3:**
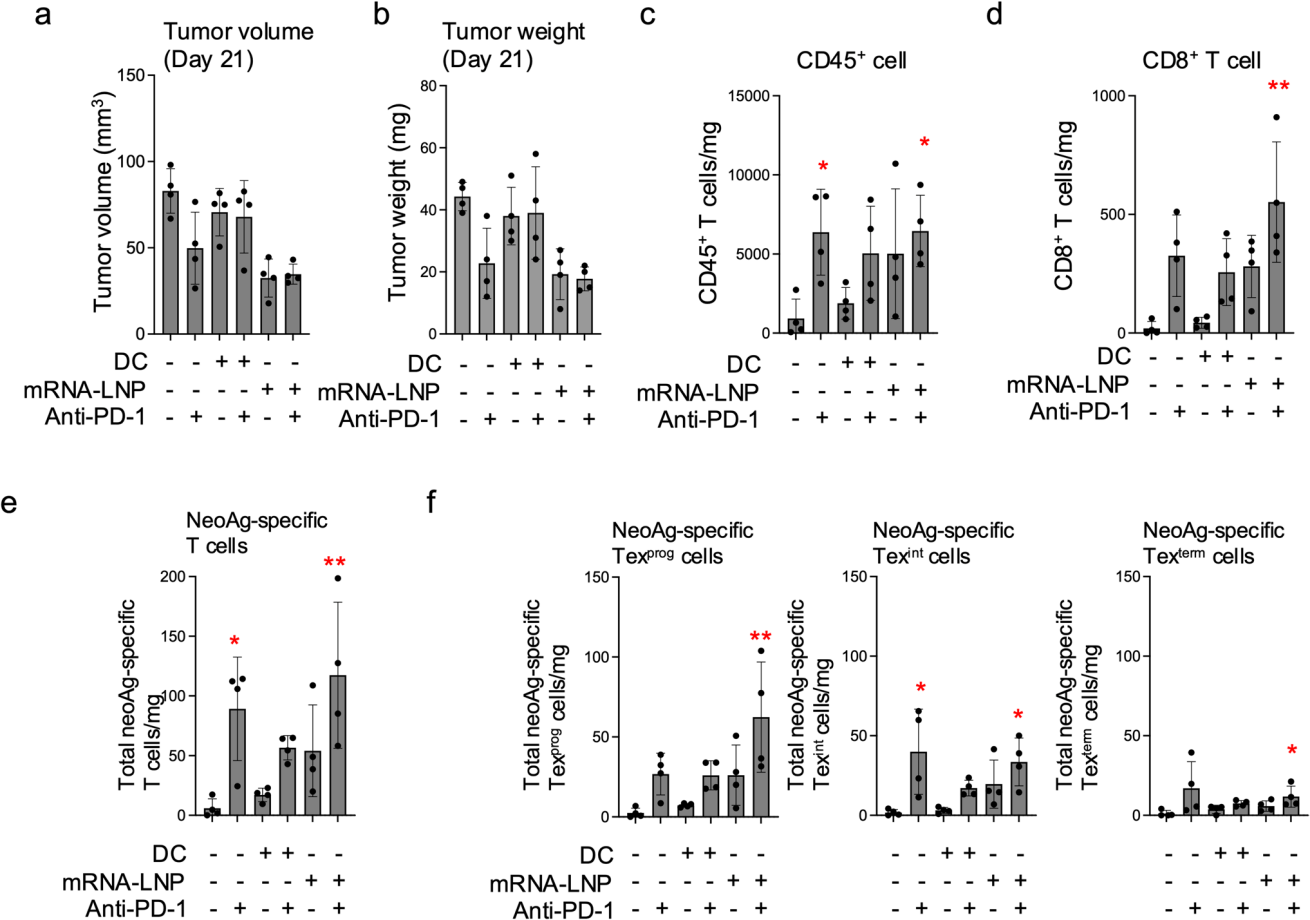
NeoAg-mRNA-LNP vaccines induced abundant neoAg-specific CD8^+^ T cell in the tumor. Mice (*n* = 4 per group) were treated as described in the legend for Fig. 2c. On day 21, the tumors were harvested and subjected to MHC class I dimer assay. **a**, **b** Tumor volume (**a**) and weight (**b**) on day 21. **c**, **d** Absolute numbers of CD45^+^ (**c**) and CD8^+^ (**d**) cells in the tumor. **e** Absolute number of total CD8^+^ T cells specific for mCdt1, mScarb2 and mZfp106 in the tumors. **f** Absolute number of total Tex^prog^ (left), Tex^int^ (center) and Tex^term^ (right) specific for mCdt1, mScarb2 and mZfp106 in the tumor. * and ** indicate *p* < 0.05 and *p* < 0.01, respectively compared to the untreated group, using the Kruskal–Wallis test followed by Dunn’s multiple comparisons test

Four days after the second treatment (day 21 post-tumor inoculation), tumor-infiltrating cells were analyzed by flow cytometry. At this time point, partial tumor regression was observed with anti-PD-1 antibody monotherapy, neoAg-mRNA-LNP monotherapy, and the combination of neoAg-mRNA-LNP with anti-PD-1 (Fig. 3a and b). The number of tumor-infiltrating CD45^+^ and CD8^+^ cells increased following treatment with the neoAg-mRNA-LNP vaccine and/or anti-PD-1 antibody, whereas no such increase was observed with the neoAg-DC vaccine (Fig. 3c and d). NeoAg-specific CD8^+^ T cells were induced within the tumor and detected in all mice, including untreated ones, using MHC class I dimer staining (Fig. 3e, Supplementary Fig. 3). A greater number of neoAg-specific CD8^+^ T cells infiltrated the tumor following neoAg-mRNA-LNP vaccination compared to neoAg-DC vaccination, and this effect was further intensified when combined with anti-PD-1 treatment (Fig. 3e).

In addition to assessing the number of neoAg-specific CD8^+^ T cells, their differentiation status was also evaluated. It is well-established that tumor-reactive T cells undergo differentiation within the tumor, progressing from a progenitor-exhausted state (Ly108⁺TIM-3⁻, Tex^prog^), which retains proliferative potential, through an intermediate-exhausted state (Ly108⁺TIM-3⁺, Tex^int^) characterized by high effector function, and ultimately into a terminally exhausted state (Ly108⁻TIM-3⁺, Tex^term^) (Supplementary Fig. 1c) [13–15]. Although a similar number of neoAg-specific CD8^+^ T cells were induced in the anti-PD-1 monotherapy group and the combination treatment group (anti-PD-1 plus neoAg-mRNA-LNP vaccination), a greater proportion of neoAg-specific CD8^+^ T cells in the combination treatment group were in the Tex^prog^ state compared to the anti-PD-1 monotherapy group (Fig. 3f). In contrast, the Tex^int^ populations were comparably elevated in both the anti-PD-1 monotherapy group and the combination treatment group compared to other groups.

These findings align with the observation that anti-PD-1 monotherapy demonstrated comparable antitumor effects on day 21 (Figs. 2c, 3a and b) but was unable to eradicate the tumors, whereas the combination treatment with anti-PD-1 and neoAg-mRNA-LNP vaccination achieved complete tumor eradication. These results suggest that the neoAg-specific Tex^prog^ population, elevated exclusively in the combination group of neoAg-mRNA-LNP and anti-PD-1, serves as a continuous source for Tex^int^ cells, thereby contributing to the sustained antitumor effect observed.

### NeoAg-mRNA-LNP vaccines prevent peritoneal dissemination of YTN16

**Fig. 4:**
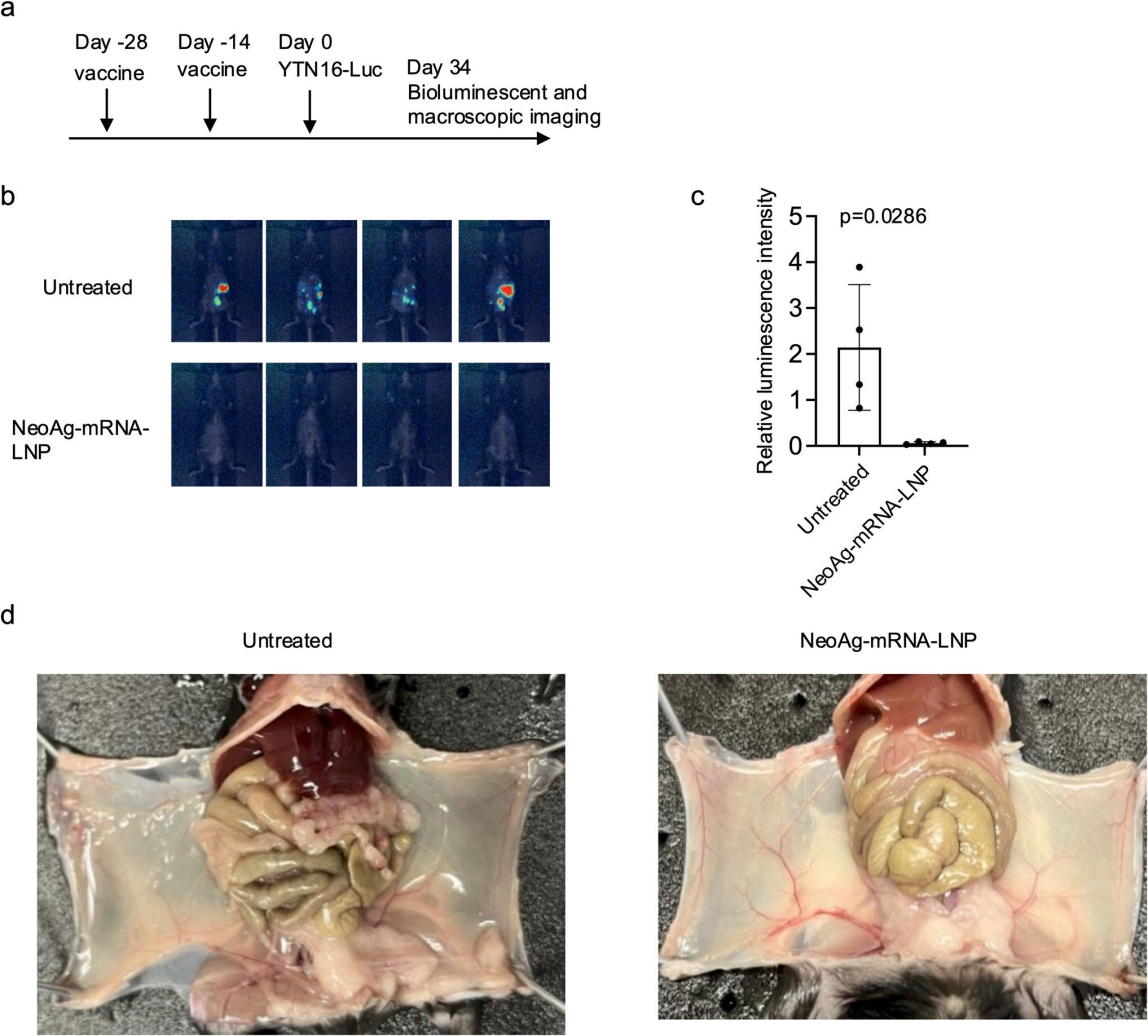
NeoAg-mRNA vaccines prevent peritoneal dissemination of YTN16. **a** Schematic representation of the experiment. Mice (*n* = 4) were vaccinated with neoAg-mRNA-LNP on days -28 and -14, followed by inoculation with 1 × 10^7^ YTN16-luciferase intraperitoneally. **b** Bioluminescent images of mice taken on day 34. **c** Relative luminescence intensities. The *p* value was calculated using the Mann–Whitney test. **d** Macroscopic images on day 34. Enlarged macroscopic images are shown in Supplementary Fig. 4

Peritoneal dissemination is a key indicator of poor prognosis in gastric cancer, occurring both as the most common form of postoperative recurrence and in patients with advanced disease. There are currently no effective treatments, and immunotherapies such as anti-PD-1 antibodies are often ineffective. To evaluate the preventive effect of the neoAg-mRNA-LNP vaccine against peritoneal dissemination of gastric cancer, mice that had received two doses of the neoAg-mRNA-LNP vaccine were intraperitoneally inoculated with YTN16 cells expressing luciferase (Fig. 4a). On day 34 after tumor inoculation, the untreated group showed clear evidence of peritoneal dissemination, confirmed by luciferase activity and macroscopic observations, while in the neoAg-mRNA-LNP-treated group, luciferase activity was barely detectable and no macroscopic signs of dissemination were observed (Fig. 4b–d and Supplementary Fig. 4). These findings suggest that the neoAg-mRNA-LNP vaccine is effective in preventing peritoneal dissemination of gastric cancer.

### Combination therapy of neoAg-mRNA-LNP vaccines and anti-PD-1 antibody exhibits a strong antitumor effect against peritoneal dissemination of YTN16 in the therapeutic setting

**Fig. 5:**
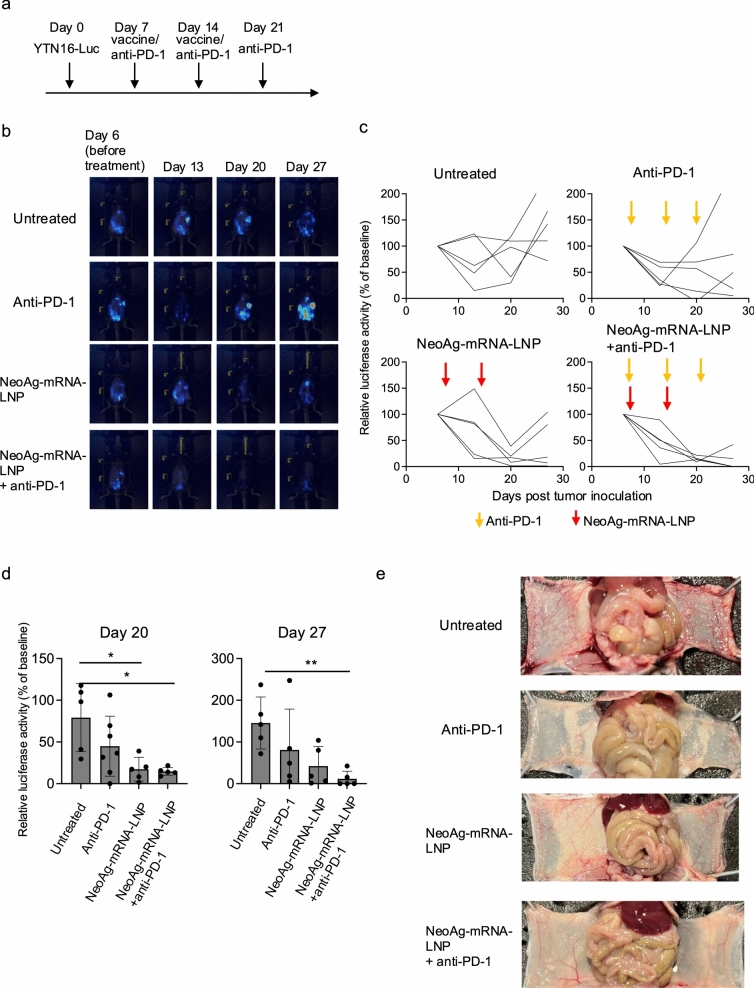
NeoAg-mRNA vaccines suppressed peritoneal dissemination of YTN16 in the therapeutic setting. **a** Schematic representation of the experiment. Mice (*n* = 5) were inoculated with 1 × 10^7^ YTN16-luciferase intraperitoneally. The mice were treated with neoAg-mRNA-LNP on days 7 and 14 and with anti-PD-1 on days 7, 14 and 21. **b** Bioluminescent images were taken on days 6, 13, 20 and 27. **c** Relative luminescent activity with day 6 as baseline (100%). **d** Relative luminescent activity on days 20 and 27. **e** Representative macroscopic images taken on day 33. Enlarged images are shown in Supplementary Fig. 5. * and ** indicate *p* < 0.05 and *p* < 0.01, respectively compared to the untreated group, using the Kruskal–Wallis test followed by Dunn’s multiple comparisons test

To evaluate the therapeutic efficacy against established peritoneal metastases, mice were inoculated intraperitoneally with YTN16 cells on day 0, followed by administration of the neoAg-mRNA-LNP vaccine on days 7 and 14, and/or anti-PD-1 antibody on days 7, 14, and 21 (Fig. 5a). In the untreated group, tumor burden, as measured by luciferase activity, was either maintained or increased over time (Fig. 5b and c). In contrast, anti-PD-1 monotherapy and neoAg-mRNA-LNP vaccine monotherapy initially led to a reduction in luciferase activity, though this was followed by a subsequent increase (Fig. 5b–d). However, in the combination therapy group, luciferase activity decreased and remained suppressed (Fig. 5b–d). Macroscopic observation on day 33 also revealed fewer metastatic lesions in the combination group compared to the untreated, anti-PD-1, and neoAg-mRNA-LNP monotherapy groups (Fig. 5e and Supplementary Fig. 5). These findings indicate that the combination of the neoAg-mRNA-LNP vaccine and anti-PD-1 antibody exhibited antitumor effects even against established gastric cancer peritoneal metastases.

## Discussion

In this study, we demonstrated that neoAg-mRNA-LNP vaccines elicit robust neoAg-specific CD8^+^ T cell responses, compared to neoAg-DC vaccines, resulting in a stronger antitumor immune response (Fig. 1). In the therapeutic setting for subcutaneous tumors, the neoAg-mRNA-LNP vaccine induced tumor regression, and its combination with an anti-PD-1 antibody further enhanced antitumor efficacy (Fig. 2). NeoAg-specific Tex^prog^ and Tex^int^ populations increased in subcutaneous tumors following combination therapy with the neoAg-mRNA-LNP vaccine and an anti-PD-1 antibody (Fig. 3). Antitumor efficacy was also observed in the peritoneal dissemination model in both prophylactic and therapeutic settings (Figs. 4 and 5).

The enhanced immunogenicity of the neoAg-mRNA-LNP vaccine over the neoAg-DC vaccine can be attributed to its ability to deliver antigen in vivo and activate endogenous DC within physiological immune niches. As shown in our previous study [16], conventional DC2 in the skin take up subcutaneously injected mRNA-LNP, express antigen encoded in the mRNA, and then migrate to the draining lymph nodes. LNP containing ionizable lipids with a vitamin E scaffold also induces type I interferon responses, promoting the upregulation of co-stimulatory molecules such as CD86 and CD40 on DC in draining lymph nodes. In contrast, ex vivo-generated DC vaccines may lack efficient homing to lymphoid tissues [17, 18]. Thus, mRNA-LNP vaccines better utilize the endogenous immune systems for efficient T cell priming. These features may be particularly beneficial in the treatment of peritoneal metastases of gastric cancer, where PD-1 blockade has shown limited efficacy.

The peritoneal metastases are the most common form of recurrence after gastric cancer surgery and are associated with poor prognosis, often accompanied by complications such as bowel obstruction and extensive ascites accumulation [2]. The introduction of immune checkpoint inhibitor (ICI) has significantly transformed the treatment paradigm for gastric cancer; however, subgroup analyses from several clinical studies indicate that ICI therapy remains insufficient for the treatment of gastric cancer patients with peritoneal metastases. The ATTRACTION-2 trial demonstrated that nivolumab monotherapy significantly improved survival outcomes in patients with advanced gastric or gastroesophageal junction cancer, showing efficacy regardless of the presence of peritoneal metastases [19]. According to the subgroup analysis of ATTRACTION-2, however, a subset of patients with peritoneal metastases, specifically those under 60 years of age, derived limited benefit from nivolumab [20]. The ATTRACTION-4 trial, which evaluated nivolumab in combination with chemotherapy in untreated, HER2-negative advanced gastric cancer, showed an extension in PFS; however, subgroup analysis indicated no efficacy in patients with peritoneal metastases [9]. These findings highlight the limitations of anti-PD-1 monotherapy or its combination with chemotherapy in certain subgroups with refractory metastatic profiles and underscore the need for alternative immunotherapeutic strategies.

Immune profiling using bulk RNA-Seq data from peritoneal metastatic tissues of gastric cancer patients classified peritoneal dissemination tissue into two groups: ‘T cell exclusive,’ where T cells were almost entirely absent, and ‘T cell exhausted,’ where T cells present expressed high levels of exhaustion markers [21]. This suggests that in peritoneal metastatic tissue, either there are few T cells, or if present, they lack the Tex^prog^ phenotype, making anti-PD-1 therapy less effective. Tex^prog^ cells play a central role in enhancing immunotherapy responses. Under chronic antigen stimulation in cancer, CD8^+^ T cells become exhausted, while Tex^prog^ cells (stem-like T cells) retain proliferative potential and can differentiate into effector Tex^int^ cells upon ICI therapy [13]. Therefore, therapies that increase tumor-reactive Tex^prog^ cells could be an effective strategy to enhance the efficacy of anti-PD-1 antibody treatment.

In our study, anti-PD-1 monotherapy led to an increase in Tex^int^ cells with effector function, but failed to increase the Tex^prog^ cells that serve as their source. As a result, although a temporary antitumor effect was observed, the antitumor activity could not be sustained, and the tumor eventually regrew due to insufficient production of sustained Tex^int^ populations. In contrast, the combination therapy of the neoAg-mRNA-LNP vaccine and anti-PD-1 significantly increased both Tex^prog^ and Tex^int^ cells, enabling a prolonged neoAg-specific T cell response capable of eradicating the tumor.

Resistance to ICI in gastric cancer patients with peritoneal metastases is also shaped by the immunosuppressive tumor microenvironment (TME) associated with peritoneal dissemination. Studies have reported that immunosuppressive CD163^+^ M2 macrophages are prevalent in peritoneal metastatic tissues and ascites from gastric cancer patients, suggesting their potential role in dampening the efficacy of ICI treatments [22, 23]. However, recent bulk RNA-Seq data from peritoneal metastatic tissues analyzed with CIBERSORT have revealed considerable variability in M2 macrophage levels among patients, suggesting the heterogeneity within peritoneal metastases-associated TME [21]. To improve therapeutic outcomes, it is crucial to elucidate the patient-specific immunosuppressive mechanisms within the peritoneal metastases-associated TME, which would allow the selection of TME-modulating agents tailored to address the unique immunosuppressive features.

In our previous study, we demonstrated that while anti-PD-1 and anti-CTLA-4 antibodies show minimal efficacy as monotherapies against YTN16 peritoneal metastases, their combination achieved partial antitumor effects in a CD8^+^ T cell-dependent manner [24]. In tumors resistant to this combination, we observed therapy-induced upregulation of the JAK-STAT pathway and increased infiltration of immunosuppressive cells, including macrophages, neutrophils, and regulatory T cells. Notably, the addition of a JAK inhibitor to combination therapy reshaped the immunosuppressive TME, leading to a potent antitumor response [24]. These findings suggest that a combination approach using neoAg-mRNA-LNP vaccines and agents that precisely modulate TME factors may provide an effective strategy for managing peritoneal metastases of gastric cancer patients.

Several clinical studies are currently investigating the combination of neoAg-mRNA vaccines with ICI. For instance, BioNTech’s autogene cevumeran (BNT122) and Moderna’s mRNA-4157 are being evaluated in solid tumors, including melanoma, lung cancer, and pancreatic cancer, often in combination with ICI agents such as atezolizumab or pembrolizumab [25]. Notably, a recent study demonstrated promising results with BNT122 when combined with atezolizumab and chemotherapy in pancreatic cancer, a malignancy traditionally difficult to treat, suggesting broader potential for neoAg-mRNA vaccines in other challenging cancers [26].

In addition to these clinical developments, the potential of neoAg-mRNA vaccines in targeting metastatic disease has also been demonstrated in preclinical and clinical studies, including those focusing on liver, lung, and lymph node metastases [26, 27]. These findings support the rationale for expanding the evaluation of such vaccines to other challenging metastatic settings, including peritoneal dissemination, as addressed in the present study.

One limitation of this study is that all experiments were conducted using a single murine gastric cancer cell line. Although this model allowed for detailed analysis of neoAg-specific immune responses in both subcutaneous and peritoneal metastasis settings, further studies using additional cell lines will be necessary to validate the generalizability of our findings.

In conclusion, this study demonstrates that neoAg-mRNA-LNP vaccines elicit strong neoAg-specific CD8^+^ T cell responses, showing significant antitumor effects in gastric cancer, particularly when combined with anti-PD-1 therapy. These findings demonstrate the potential of neoAg-mRNA vaccines to enhance immunotherapy efficacy and offer a promising new strategy for targeting advanced gastric cancer with peritoneal dissemination.

## Supplementary Information

Below is the link to the electronic supplementary material.

Supplementary file1 (PDF 1279 KB)

Supplementary file2 (PDF 22 KB)

Supplementary file2 (XLSX 22 KB)
