# Supplementary material for: Neoantigen mRNA vaccines induce progenitor-exhausted T cells that support anti-PD-1 therapy in gastric cancer with peritoneal metastasis
Source: Gastric Cancer. 2025 Jul 31;28(5):825–36. doi: 10.1007/s10120-025-01640-8 (PMC12378472; doi:10.1007/s10120-025-01640-8)
Supplement: Supplementary file 1 — Supplementary file1 (PDF 1279 KB) [file 10120_2025_1640_MOESM1_ESM.pdf]

Supplementary Figure 1

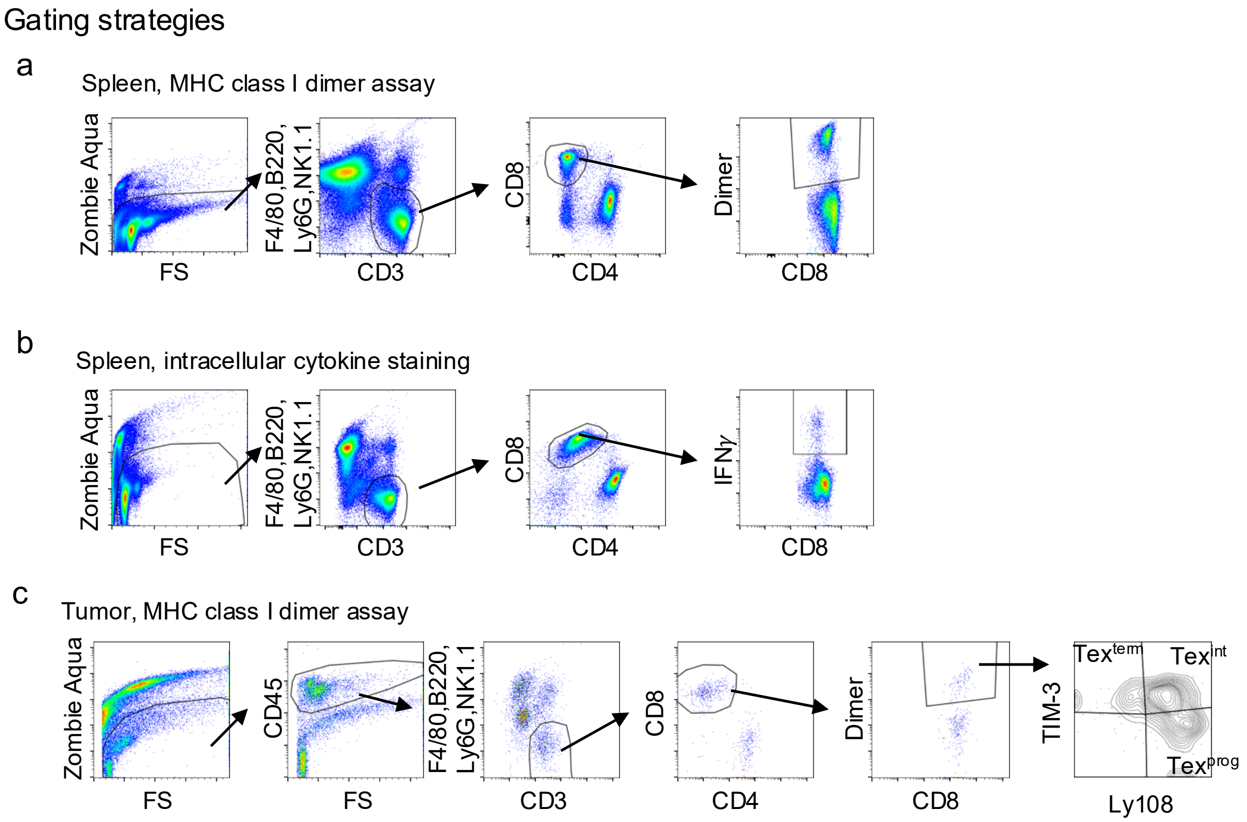

Supplementary Figure 1. Gating strategies.
Gating strategies for the MHC dimer assay of spleen (a), intracellular cytokine staining (b) and MHC class I dimer assay of tumor (c).

Supplementary Figure 2

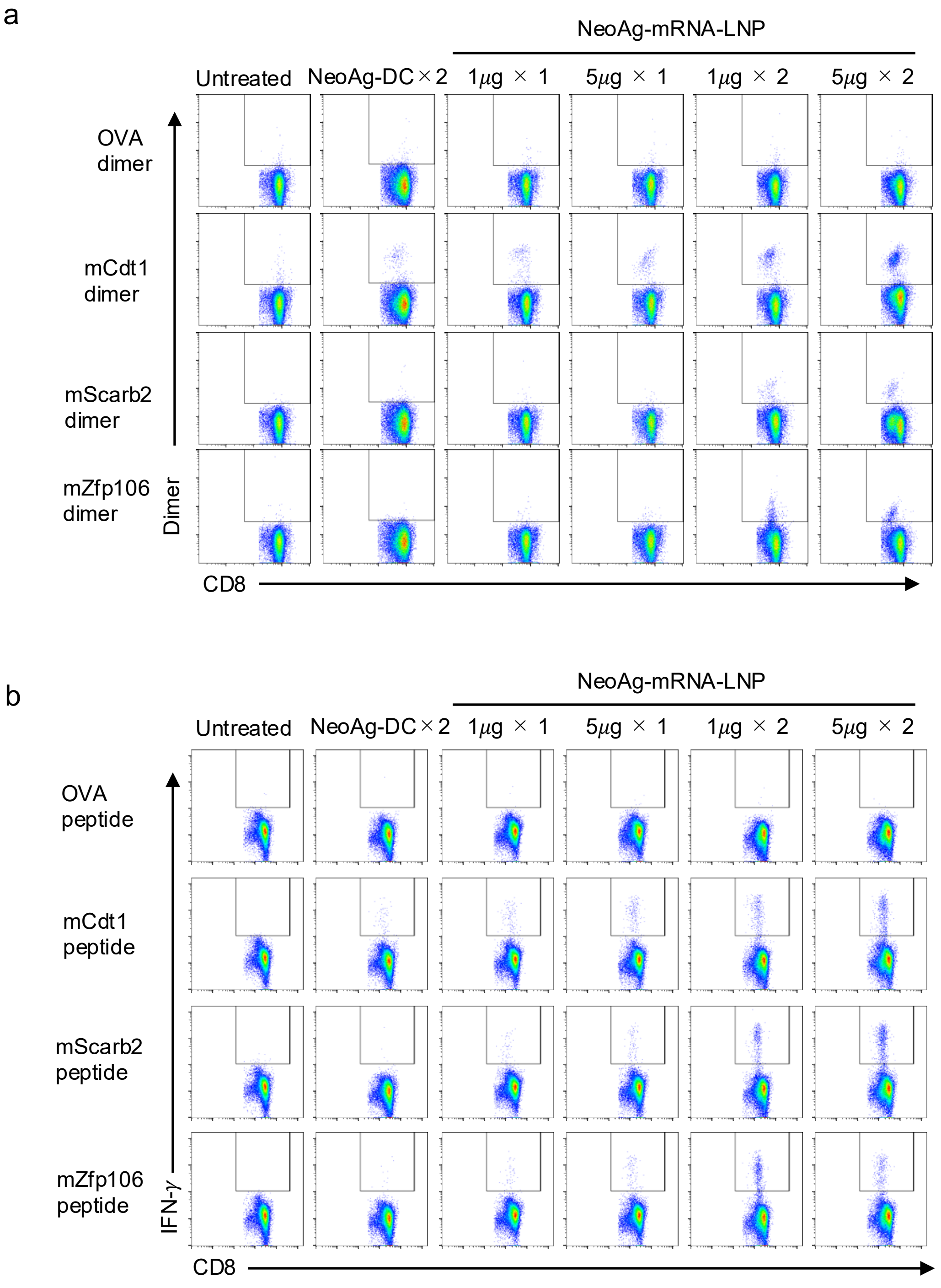

Supplementary Figure 2. Neoantigen mRNA-LNP vaccines efficiently induce neoantigen-specific CD8⁺ T cells.
Naïve mice (n=4 or 5 per group) were treated as described in the legend for figure 1a and b. (a,b) Representative dot plots corresponding to the data in Figure 1a (a) and Figure 1b (b).

Supplementary Figure 3

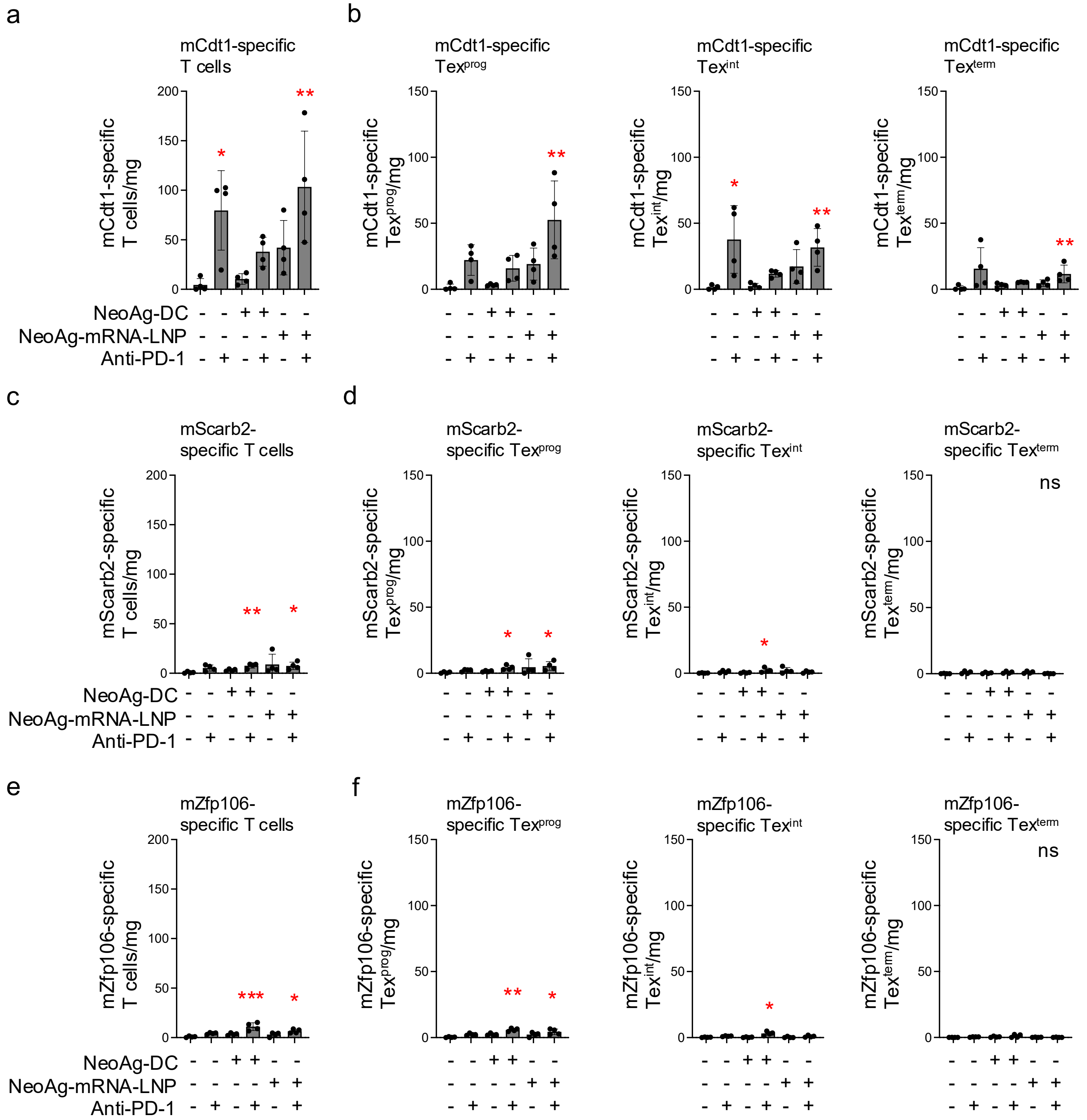

Supplementary Figure 3. NeoAg-mRNA-LNP vaccines induced abundant neoAg-specific CD8$^+$ T cell in the tumor.
Mice were treated as described in the legend for Figure 3. (a, b) Absolute number of mCdt1-specific CD8$^+$ T cells (a), mCdt1-specific Tex^{prog} (b, left), Tex^{int} (b, center) and Tex^{term} (b, right) in the tumor. (c, d) Absolute number of mScarb2-specific CD8$^+$ T cells (c), mScarb2-specific Tex^{prog} (d, left), Tex^{int} (d, center) and Tex^{term} (d, right) in the tumor. (e, f) Absolute number of mZfp106-specific CD8$^+$ T cells (e), mZfp106-specific Tex^{prog} (f, left), Tex^{int} (f, center) and Tex^{term} (f, right) in the tumor. * indicates $p < 0.05$, compared to the untreated group, using the Kruskal-Wallis test followed by Dunn's multiple comparisons test. ns; $p>0.05$.

Supplementary Figure 4

Untreated

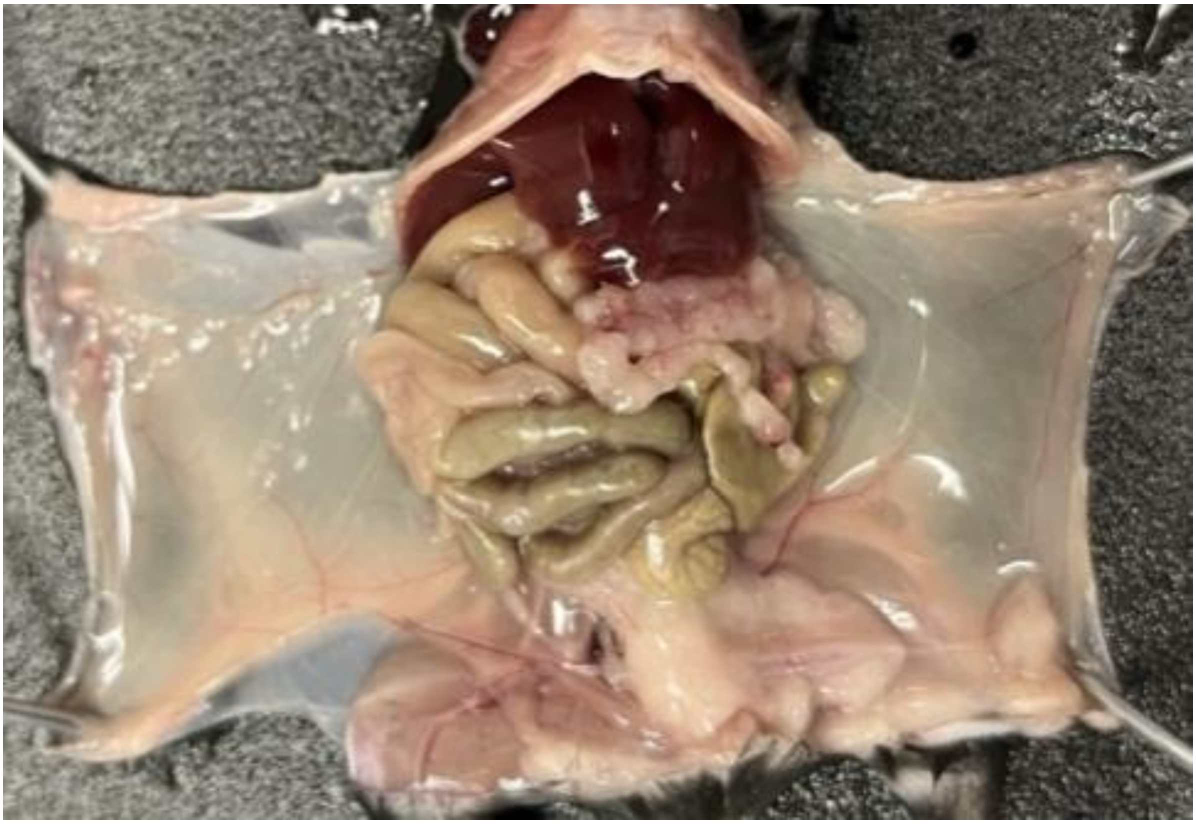

NeoAg-mRNA-LNP

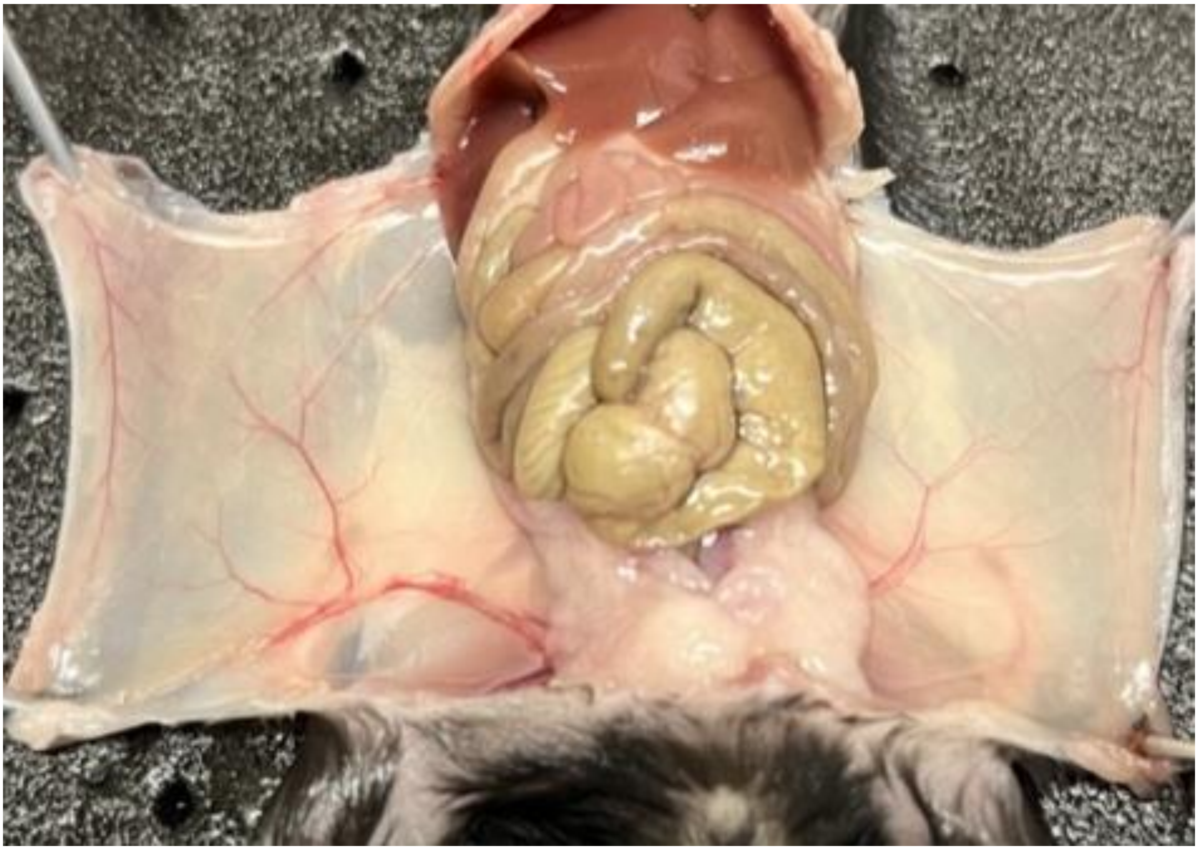

Supplementary Figure 4. Enlarged image of Figure 4d.
This supplementary figure presents an enlarged version of the image shown in Figure 4d.

Supplementary Figure 5

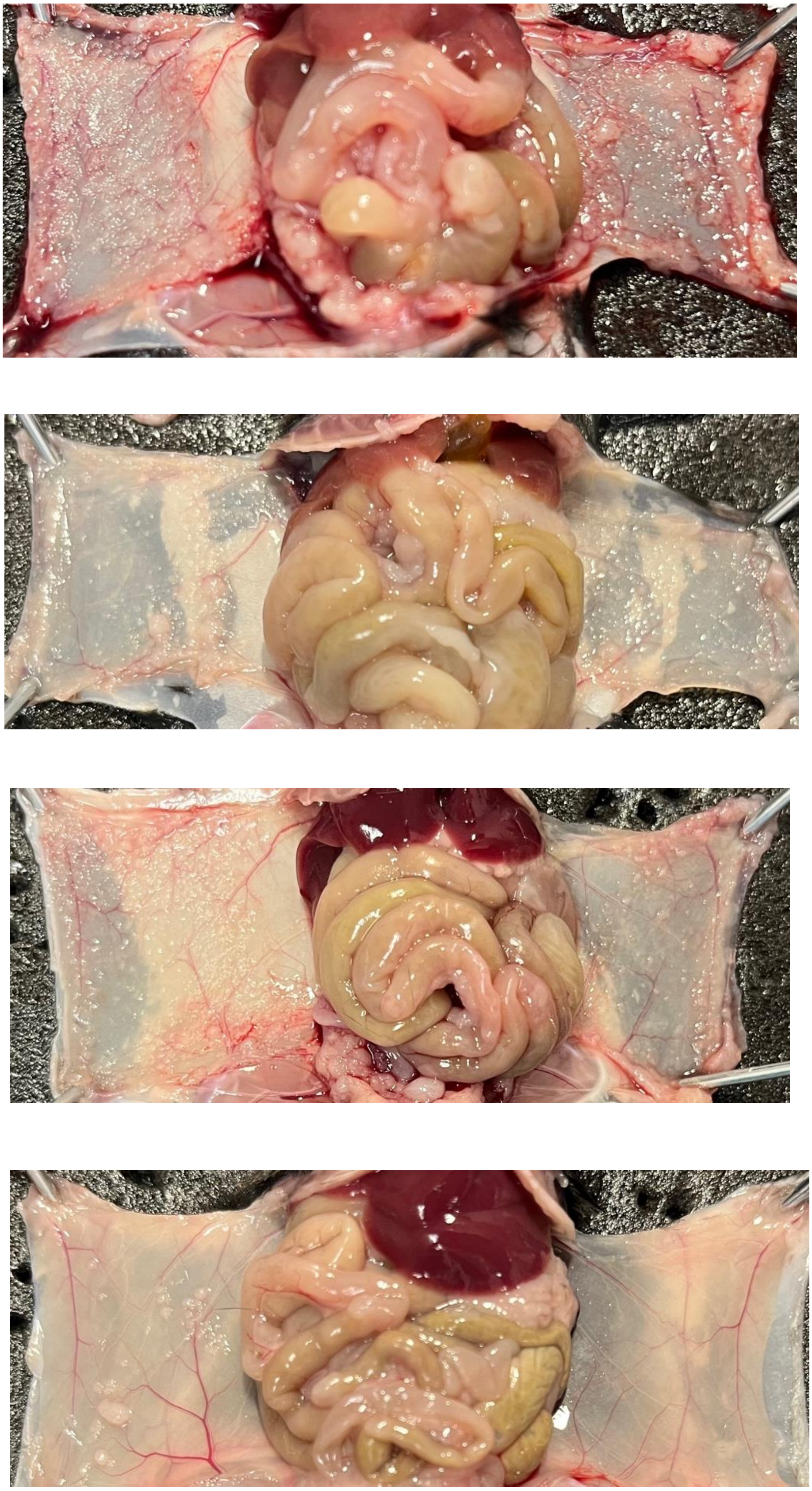

Supplementary Figure 5. Enlarged image of Figure 5e.
This supplementary figure presents an enlarged version of the image shown in Figure 5e.
