# Supplementary material for: Neoantigen mRNA vaccines induce progenitor-exhausted T cells that support anti-PD-1 therapy in gastric cancer with peritoneal metastasis
Source: Gastric Cancer. 2025 Jul 31;28(5):825–36. doi: 10.1007/s10120-025-01640-8 (PMC12378472; doi:10.1007/s10120-025-01640-8)
Supplement: Supplementary file 2 — Supplementary file2 (PDF 22 KB) [file 10120_2025_1640_MOESM2_ESM.pdf]

Supplementary Materials and Methods

Establishment of YTN16-luciferase (YTN16-Luc) cells.

The firefly luciferase gene from pCMVluc+ (Riken BRC) was subcloned into the pCXN2 plasmid. The plasmid was transfected into YTN16 cells using Lipofectamine 2000 (ThermoFisher Scientific), following the manufacturer's protocol. After selection with G418 and limiting dilution, luciferase-expressing clones were selected by adding D-luciferin and measuring luminescence using the TriStar LB 941 Multimode Microplate Reader (Berthold Technologies).

Preparation and purification of neoAg-mRNA

The neoAg-coding DNA fragment was synthesized by GeneArt Strings DNA fragments synthesis service (ThermoFisher Scientific) and cloned into a plasmid DNA (pDNA) vector containing the 5'- and 3'-untranslated region (UTR) sequences. The template DNA for *in vitro* transcription was prepared by PCR using the neoAg-coding pDNA as a template, PrimeSTAR MAX DNA polymerase (Takara Bio, Shiga, Japan), a forward primer containing the T7 promoter sequence, and a reverse primer containing the 120-mer poly(T) sequence. *In vitro* transcription from the obtained template DNA was performed using either the MEGAscript T7 Transcription Kit (ThermoFisher Scientific) or Takara IVTpro T7 mRNA Synthesis Kit (Takara Bio). N1-methylpseudouridine-5'-triphosphate (TriLink Biotechnologies, San Diego, CA, USA or FUJIFILM Wako Pure Chemical Corporation, Osaka, Japan) was used instead of UTP for the complete substitution of uridine with N1-methylpseudouridine. CleanCap Reagent AG (TriLink Biotechnologies) was used for the co-transcriptional capping of mRNAs. The transcribed mRNA was purified using RNAClean XP (Beckman Coulter Inc., Brea, CA, USA) and dephosphorylated using Quick CIP (New England Biolabs Japan Inc., Tokyo, Japan). The dephosphorylated mRNA was then purified using RNeasy Mini Kit (Qiagen K.K., Tokyo, Japan). The concentration of purified mRNA was measured using Nanodrop ONE (ThermoFisher Scientific). The length of

mRNA was analyzed using an Agilent 2100 Bioanalyzer and Agilent RNA6000 Nano Kit (Agilent Technologies Japan Ltd., Tokyo, Japan).

Preparation of neoAg-mRNA-LNP

Preparation of mRNA-LNP was conducted based on post-encapsulation method (manuscript under review). The lipid molecules, COATSOME® SS-OP, COATSOME® SS-EC, dioleoyl-sn-glycero phosphatidyl choline (DOPC), and 1,2-dimyristoyl-rac-glycero-3-methoxypolyethylene glycol-2000 (DMG-PEG_{2000}) were purchased from NOF CORPORATION. Cholesterol was purchased from Sigma-Aldrich. The empty LNP was prepared by mixing the lipid mixtures with composition of SS-OP/SS-EC/DOPC/cholesterol = 32.5/20/7.5/40 (mol% of total lipid) with additional DMG-PEG_{2000} (1.5 mol% of total lipid). The lipid mixtures were dissolved in ethanol to a concentration of 8.0 mM (total lipid in ethanol). The lipid mixtures in ethanol and 20 mM malic acid buffer (pH 3.0; salt free) were set to their respective syringe into NanoAssemblr® Ignite™ device (Precision Nanosystems, Vancouver, Canada) with the setting of flow rate = 16.0 mL/min and flow rate ratio (buffer: lipid) = 7 : 1. The resulting suspension of LNP in the mixture of ethanol and malic acid buffer was diluted with 20 mM MES buffer (pH 6.0) for at least 4-fold and transferred into Amicon Ultra-4-100K Centrifugal Units (Merck, Rahway, NJ, USA). Centrifugation (1000×*g*, room temperature (RT)) was done to concentrate the LNPs suspension. The resulting LNP remaining in the upper cassette was re-diluted with the 20 mM MES buffer (pH 6.0) for at least 10-fold and centrifuged again (1000×*g*, RT). The LNPs suspension were then collected and diluted to an adequate volume with 20 mM MES buffer (pH 6.0) to adjust the concentration to 40 mM of LNP. An equal volume of 320 mg/mL of sucrose solution (nacalai tesque, Kyoto, Japan) was mixed well into the LNP suspension. The post-encapsulation of neoAg-mRNA was done by mixing 100 nmol of LNPs with 0.006 µg/µL neoAg-mRNA solution in 20 mM MES buffer (pH 6.0). The neoAg-mRNA-LNPs were mixed well followed by heating at 37 °C for 5 minutes, then allowed to cool (RT) for approximately 10 minutes. The neoAg-mRNA-

LNPs were then diluted with 50 mM Tris buffer (pH 7.4; 150 mM NaCl) and transferred into Amicon Ultra-4-100K Centrifugal Units (Merck, Rahway, NJ, USA). Centrifugation (1000×*g*, RT) was done to concentrate the LNPs suspension. The neoAg-mRNA-LNPs were collected and an adequate volume of 50 mM Tris buffer (pH 7.4; 150 mM NaCl) was added to adjust the concentration into 100 mg/mL mRNA.

Preparation of neoAg-DC vaccines

Dendritic cells (DC) were prepared as described previously (1). Briefly, bone marrow cells from femurs and tiblas were cultured for 8 days in RPMI1640 supplemented with 10% FBS, 10mM HEPES, 1mM sodium pyruvate, 1mM MEM nonessential amino acid solution, 5mM 2-mercaptoethanol, 100U/mL penicillin, 100 μg/mL streptomycin and 20 ng/mL mouse GM-CSF (PeproTech, Rocky Hill, NJ, USA). Then DC were stimulated with 1 μg/mL lipopolysaccharide (FujiFilm Wako Pure Chemical Corporation) for 16 hours and separately pulsed with mCdt1, mScarb2 and mZfp106 peptides at 1 μg/mL for 2 hours. After washing, equal number of 3 different peptides-pulsed DC were pooled and injected subcutaneously.

Peptide loading onto MHC class I dimers

For the preparation of MHC class I dimers, 0.375 μg of each peptide (H-2K^b-mCdt1 (KTVYPMSYRF), H-2K^b-OVA (SIINFEKL), H-2D^b-mScarb2 (TSVINTTLV), H-2D^b-mZfp106 (TSPRNSTVL), and H-2D^b-hgp100 (KVPRNQDWL)) was incubated with 0.5 μg of DimerX I: Recombinant Soluble Dimeric Mouse H-2K^b or DimerX I: Recombinant Soluble Dimeric Mouse H-2D^b (both from BD Biosciences) at 37°C overnight. The peptide-loaded MHC dimers were then mixed with 0.5 μg of PE-conjugated anti-mouse IgG1 monoclonal antibody (clone RMG1-1, BioLegend, San Diego, CA, USA) and incubated for 60 minutes at room temperature. This was followed by an additional 30-minute incubation with purified mouse IgG1 isotype control monoclonal antibody (clone MOPC-21, BioLegend) at room temperature.

Flowcytometry

For preparation of tumor-infiltrating cells, tumor tissues were cut into pieces and incubated in RPMI1640 supplemented with 0.2% collagenase (FujiFilm Wako Pure Chemical Corporation) and 2 KU/ml DNase I (Sigma-Aldrich) for 40 min at 37 °C, passed through a 70 µm cell strainer. For intracellular cytokine staining, splenocytes (1×10^6) were cultured for 4 hours with the indicated peptides at 1 µg/ml or with 1×10^5 YTN16 cells treated with mouse IFN-γ (10 U/ml, Peprotech, Cranbury, NJ, USA) in the presence of 10 µg/ml brefeldin A (Sigma-Aldrich, St. Louis, MO, USA). After staining dead cells with the Zombie Aqua Fixable Viability Kit, blocking Fc receptors with anti-CD16/32 mAb, and staining with mAbs for cell surface antigens, the cells were fixed and permeabilized with Fixation Buffer (BioLegend) and Intracellular Staining Perm Wash Buffer (BioLegend) according to the manufacturers' protocols, followed by staining with mAbs for IFN-γ. For MHC class I dimer assay, the cells were stained with the MHC class I dimer complex for 2 hours. Subsequently, the cells were stained with mAbs against cell surface antigens. The stained cells were acquired on a CytoFLEX flow cytometer (Beckman Coulter, Brea, CA, USA) and the data were analyzed using FlowJo software (FlowJo, LLC, Ashland, OR, USA). Gating strategies are shown in Supplementary Figure 1.

Sample Size Determination

Based on our previous experience and in accordance with the 3Rs principle (Replacement, Reduction, and Refinement), the sample size for animal experiments was determined using G*Power software (Version 3.1.9.6). To ensure the ethical use of animals while maintaining statistical validity, we performed a power analysis using the ANOVA: Fixed effects, omnibus, one-way test. The parameters were adjusted based on the specific experimental conditions: effect size $f = 0.7–0.8$ and statistical power $1-\beta = 0.7–0.8$, with a significance level of $\alpha = 0.05$. These settings were selected to balance reducing the number of animals while ensuring sufficient power

to detect meaningful differences between groups, depending on the experiment's requirements.

Reference

1. Nagaoka K, Hosoi A, Iino T, Morishita Y, Matsushita H, Kakimi K. Dendritic cell vaccine induces antigen-specific CD8(+) T cells that are metabolically distinct from those of peptide vaccine and is well-combined with PD-1 checkpoint blockade. Oncoimmunology. 2018;7(3):e1395124.
